# Intestinal and vascular smooth muscle relaxant effect of *Viscum album* explains its medicinal use in hyperactive gut disorders and hypertension

**DOI:** 10.1186/s12906-016-1229-3

**Published:** 2016-07-27

**Authors:** Taous Khan, Sayyad Ali, Rahila Qayyum, Izhar Hussain, Fazli Wahid, Abdul Jabbar Shah

**Affiliations:** 1 Department of Pharmacy, COMSATS Institute of Information Technology, Abbottabad, 22060 Pakistan; 2 Department of Environmental Sciences, COMSATS Institute of Information Technology, Abbottabad, 22060 Pakistan

**Keywords:** Viscum album, Fraction, Rabbit jejunum and aorta, Gut spasm, Vasorelaxation, Ca^++^ antagonist

## Abstract

**Background:**

*Viscum album* has shown inhibitory effect on different smooth muscles but underlying mechanisms in gut and vascular smooth muscles are not well defined. Additionally, the plant has also importance in managing hyperactive gut and cardiovascular disorders. The current study was aimed to probe a pharmacological base of the smooth muscle relaxant effect of *V. album* in gut and vascular preparations.

**Methods:**

*V. album* crude extract (Va. Cr) and its ethyl acetate fraction (Va. EtAc) were studied using in vitro techniques. The antispasmodic activity was performed using isolated rabbit jejunum while the vasorelaxant effects were studied in rabbit aortic rings.

**Results:**

Va. Cr and Va. EtAc inhibited spontaneous and high K^+^-induced contractions with EC_50_ values of 0.31 mg/mL (0.15–0.57) and 0.62 mg/mL (0.3–0.95), respectively. This advocates an antispasmodic effect probably operated through calcium channels blockade (CBB). The proposed mechanism was confirmed by a pretreatment of the tissue with Va. Cr (0.01–0.3 mg/mL), which shifted the Ca^++^ concentration-response curves (CRCs) rightward, similar to verapamil. Moreover, Va. Cr showed a partial relaxation against high K^+^ and PE (1 μM) induced contractions in isolated rabbit aorta rings. Va. EtAc caused complete relaxation of high K^+^ precontraction and partially relaxed PE (1 μM) induced contractions, suggesting inhibitory effect on Ca^++^ entry, in addition to other possible mechanisms. CRCs were shifted to the right correspondingly to verapamil when the aortic rings were pretreated with Va. Cr and Va. EtAc.

**Conclusions:**

These data indicated that Va. Cr possesses smooth muscle relaxant effect mediated through voltage-dependent Ca^++^ channel blockade (CCB), which explains its spasmolytic and vasorelaxant activity. The CCB activity is concentrated more in Va. EtAc. This study provides an evidence for the medicinal importance of *V. album* in gut spasm and possibly hypertension.

## Background

*Viscum album* belongs to the family Loranthaceae. It is a small greenish plant, locally named as “guch” or “ghwar” and mistletoe in English [1]. It is a hemiparasite on other plants like wall nut or apricot. The plant grows in Northern areas of Pakistan frequently on the hills in Chitral and Dir Upper [2]. The plant can grow up to 100 cm with leathery leaves alternate on the stems. Fruit of *V. album* is waxy white gelatinous berry [3]. Medicinal use of *V. album* can be found in ancient Greeks practices [4]. It has been documented that Hippocrates (460–377 BC) used *V. album* for the treatment of spleen diseases and ailments related with menstruation [5]. According to the Platonist Celsus, *V. album* was used around 150 AD for the treatment of swellings or tumors [5]. In the third quarter of the 1^st^ century AD, a priest Druids used it to treat almost every illness or “all-heal”, and even as an antidote for poisons and treating infertility [5]. Later on, *V. album* was practiced for the treatment of epilepsy and used as a remedy for seizures until the 18^th^ century [5]. *V. album* is used in folklore medicines as a remedy for various diseases, such as neuralgia, sciatica, epilepsy, rheumatic deafness and bronchial asthma [1–3], diabetes mellitus, chronic cramps, stroke, stomach problems, as antihypertensive and for hot flush in menopause [6]. Medicinally, *V. album* is documented as diuretics, antispasmodic and useful in cardiac problems [7].

Various chemical constituents reported from *V. album* include alkaloids, glycosides, phenylpropanoids, tannins, lignins sugars and viscotoxins [8]. It also contains various flavonoids including quercetin, kaempferol and rarely naringenin [9]. Methyl derivatives of quercetin and kaempferol have also been reported from this plant [9].

Literature shows extensive studies on either the extract and or chemical constituents isolated from *V. album* in different types of cancer, including bladder [10], breast [11], pancrease [12] and many more. However, limited studies are available, which have shown that extract and compounds isolated from *V. album* attenuated smooth muscle activity [13]. The contraction of smooth muscle preparations, including rabbit jejunum and aorta is dependent upon an increase in the cytoplasmic free [Ca^++^], which activates the contractile elements [14]. The increase in intracellular Ca^++^ occurs either via influx through voltage-dependant Ca^++^ channels (VDCs) or its release from intracellular stores. Periodic depolarization and repolarization regulates the spontaneous movements of the intestine and at the height of depolarization, the action potential appears as a rapid influx of Ca^++^ via VDCs [15]. The increase in the intracellular Ca^++^ is also responsible for the increase in vascular resistance and blood pressure.

We hypothesized that the inhibitory effect of *V. album* on smooth muscles might be due to constituents having inhibitory effect on Ca^++^ influx through voltage-dependent Ca^++^ channels. Because a Ca^2+^ increase in smooth muscle is required for contraction and calcium antagonists induce relaxation of both intestinal and vascular smooth followed by a consequent reduction in intestinal motility and blood pressure [16]. Therefore, an indirect approach was adopted to investigate the antispasmodic and vasorelaxant potential of the extract of *V. album*, using in vitro pharmacological approach to probe the underlying mechanisms of action.

## Methods

### Plant material

The fruit of the plant was collected from distract Dir Upper, near Sheringal in November 2012. It was identified and authenticated by Prof. Dr. Manzoor Husain and Assistant Prof. Dr. Mujtaba, Post Graduate College No.1 Abbottabad, Pakistan. The voucher specimen (Nov12 Va1) was placed at the herbarium of that institute.

### Preparation of crude extract and fractions

The fruit was first freed from adulterants by washing with tap water and then shade dried at ambient temperature. The dried plant material was pulverized to powder and a methanolic extract was prepared through cold maceration process. For this purpose, the powdered plant material (4.4 kg) was soaked in methanol for 15 days with occasional shaking. After this, it was first passed through a muslin cloth followed by filtration through a Whatman filter paper [17]. The same process was repeated 2 more times. All the filtrates were pooled together and evaporated in a rotary evaporator (Heidolph HB Digital Laboratories, Germany) under reduced pressure at 45 °C. The final crude extract (Va. Cr) was obtained as a thick dark green semi-solid mass (31 % yield).

In order to obtain subsequent fractions, Va. Cr was dispersed in sufficient quantity of distilled water and successively shaken with *n*-hexane, chloroform, ethyl acetate and n-butanol in ascending order of polarity. All the fractions were individually evaporated in a rotary evaporator (Heidolph HB Digital Laboratories, Germany) under reduced pressure [18]. All the subsequent fractions obtained from the crude extract were subjected to the preliminary screening for evaluation of their relaxing effect on jejunal and aortic tissues. Only the ethyl acetate fraction (Va. EtAc) showed significant activity due to which it was selected for further detailed bioactivity studies.

### Preliminary phytochemical analysis

Va. Cr and Va. EtAc were screened for the presence of various phytoconstituents including tannins, alkaloids, saponins, phenolic compounds, cardiac glycosides, anthraquinone glycosides, sapoinns, flavonoids, steroids and terpenes by using standard protocols previously described [19].

Test for tannins and phenolic compounds was carried out by mixing 20 mg sample with 10 ml of distilled water followed by heating. Then few drops of 1 % ferric chloride solution were added. The appearance of a blue-black, green or blue-green color indicated the presence tannins and phenolic compounds. Alkaloids were tested by shaking each sample (20 mg) with 5 ml HCl (1 %) followed by gentle heating in a water bath for 1 min. To this mixture, few drops of Dragendorff’s reagent was added. Formation of orange-red precipitates showed the presence of alkaloids. The presence of saponins was detected by using froth test. Each sample (40 mg) was vigorously shaken with 5 ml of distilled water for 5 min. Formation of a persistent froth on warming indicated the presence of saponins. Cardiac glycosides were detected by adding 1 ml of acetic acid to the test samples (100 mg) in a test tube. The solution was cooled well in ice and then concentrated H_2_SO_4_ was carefully added. Formation of a bluish green precipitates indicated the presence of cardiac glycosides. To test the anthraquinone glycosides, aqueous ammonia (10 % v/v) solution was slowly added to the sample solution in benzene and then shaken. A change in color to red, violet or pink indicated the presence of anthraquinones. For steroids detection, a solution consisting of 2 ml chloroform, 1 ml sulphuric acid, and 1 ml of acetic acid was prepared and then gradually added to the sample solution. The appearance of greenish color indicated the presence of steroids. For terpenoids, a small amount of the test sample was dissolved in ethanol. Then 1 ml of acetic anhydride was added to it followed by the addition of concentrated H_2_SO_4_. A change in colour from pink to violet indicated the presence of terpenoids. For flavonoids detection, 2 ml of the 10 % aqueous sodium hydroxide was added to the aqueous solution of the test sample. This resulted in the production of a yellow colouration. A change in colour from yellow to colourless on addition of dilute HCl solution was an indication for the presence of flavonoids.

### Drugs and standards

The chemicals and drugs including verapamil hydrochloride, phenylephrine, acetylcholine chloride, potassium chloride and calcium chloride were purchased from Sigma Chemical Company (St. Louis, MO, U.S.A.) and were of maximum purity. Stock solutions were prepared in distilled water while their dilutions were freshly prepared at the time of experiments using normal saline as vehicle.

### Animals

Handling, care and use of animals was carried out according to the rules of the Institute of Laboratory Animal Resources, Commission on Life Sciences, National Research Council (National Research Council, 1996). All experiments were performed with prior approval from the Ethical Committee of the COMSATS Institute of Information Technology, Abbottabad, Pakistan, in its meeting held on 17–06–2013 vide notification EC/PHM/07–2013/CIIT/ATD. Local breed rabbits of either sex weighing 1.5–2 kg were used in the study and were kept under controlled conditions (23–25 °C) in the animal house of COMSATS Institute of Information Technology, Abbottabad. Animals received a standard diet and had a free access to water.

### Rabbit jejunum

The effects of Va. Cr and Va. EtAc on rabbit jejunum were determined according to protocols as described previously [20]. The rabbits were fasted for 24 h before the experiment but a free access to water was permitted. The animals were executed by cervical dislocation followed by dissection of the abdomen and isolation of the jejunum. Jejunum segments of about 2 cm length were suspended in a 10 mL tissue baths filled with Tyrode’s solution constantly kept at 37 °C and bubbled with carbogen (5 % carbon dioxide in oxygen). Tyrode’s solution was composed of (mM): glucose 5.6, NaCl 136.9, KCl 2.7, MgCl_2_ 1.1, NaH_2_PO_4_ 0.4, NaHCO_3_ 11.9 and CaCl_2_ 1.8 (pH 7.4). After application of 1 g preload, the tissues were kept uninterrupted for 30 min of equilibrium period. Then the control responses were obtained to a sub-maximal concentration of acetylcholine (0.3 μM). The tissue was considered stable when reproducible responses were obtained.

Rabbit jejunum was used to find out the calcium channel blocking (CCB) activity of Va. Cr and Va. EtAc. For this purpose, pre-contraction of the tissue was carried with a high concentration (80 mM) of K^+^ [21]. After obtaining a plateau, samples were applied in a cumulative mode in order to get concentration-dependent inhibitory response curves [22]. The relaxant effect of the samples was calculated with reference to the control response obtained with K^+^. To further confirm the Ca^++^ antagonist activity of Va. Cr and Va. EtAc, the tissue was stabilized for 30 min in normal Tyrode’s solution. Then this solution was substituted with a Ca^++^-free Tyrode’s solution containing ethylenediaminetetraacetic acid (EDTA, 0.1 mM) in order to eliminate the Ca^++^ from the tissue bath. This solution was also substituted with Ca^++^-free and K^+^-rich Tyrode’s solution containing (mM): glucose 5.55, NaCl 91.04, KCl 50, MgCl_2_ 1.05, NaH_2_PO_4_ 0.41, NaHCO_3_ 11.87 and EDTA 0.1. The tissue was incubated for 30 min in this solution after which reproducible cumulative CaCl_2_ concentration response curves were obtained for Va. Cr, Va. EtAc and verapamil. All data were recorded and analyzed with the help of a force transducer coupled with a bridge amplifier data acquisition system (AD Instruments, Sydney, Australia).

### Rabbit thoracic aorta

The vasorelaxant activity of Va. Cr and Va. EtAc was determined by following the previously reported protocols [23, 24]. The thoracic aorta was isolated after execution of rabbits by cervical dislocation. Then it was cut into rings of about 2–3 mm width. The preparations were then mounted by use of a pair of stainless steel hooks in a 5 mL organ bath. One hook was attached to a steel rod at the bottom while the other one was attached to a force transducer (MLT 0201). The tissue bath contained normal Kreb’s solution that was composed of (mM): NaCl 118.2, NaHCO_3_ 25.0, CaCl_2_2.5, KCl 4.7, KH_2_PO_4_ 1.3, MgSO_4_ 1.2 and glucose 11.7 (pH 7.4). This solution was constantly maintained at 37 °C and aerated with carbogen (5 % CO_2_ in O_2_). After application of a resting tension of 2 g, the tissue was equilibrated for 1 h before studying the effect of the samples. Stabilization of the preparations was achieved with repeated concentrations of phenylephrine (PE, 1 μM). The data (changes in isometric tension) were recorded and analyzed with the help of a force transducer coupled with a bridge amplifier data acquisition system (AD Instruments, Sydney, Australia).

### Effect on contraction induced by phenylephrine and high K^+^

The previously described protocol [25] was followed for this purpose with slight modifications. Steady-state contractions of tissue were induced with phenylephrine (1 μM) or high K^+^. In order to obtain concentration response relationship the Va. Cr and Va. EtAc were cumulatively added to the tissue bath. The relaxation was calculated with reference to percent of agonist-induced contractions. The vascular reactivity of the samples was determined on Ca^++^ influx through voltage-dependent channels.

### Determination of calcium channel blocking activity

In this case, the control CRCs of Ca^++^ (as CaCl_2_) were obtained after washing of the aortic rings with Ca^++^-free solution 4 to 5 times. After obtaining superimposable (usually after 2 cycles) control CRCs of Ca^++^, the tissue was pretreated with Va. Cr and Va. EtAc for 30–45 min to determine the possible calcium channel blocking activity. A control experiment was also performed using the same experimental conditions [26].

### Data analysis

Wherever needed statistical analysis was applied. The data given are expressed as ± standard error means (SEM). The median effective concentrations (EC_50_ values) are given with 95 % confidence intervals. Student’s *t*-*test* was applied with *p* < *0.05* noted as significantly different.

## Results

### Phytochemical analysis

Both Va. Cr and Va. EtAc were analyzed for the presence of various groups of chemical constituents and the results have been displayed in Table 1. Both samples were standardized by using standard methods and found to be very rich in tannins, alkaloids, saponins, phenols, cardiac glycosides, flavonoids, steroids, anthraquinone glycosides and terpenoids (Table 1).

**Table 1:** Phytochemical analysis of the crude extract of *Viscum album* and its ethyl acetate fraction

Constituents	Crude extract	Ethyl acetate fraction
Tannins	+ + +	+++
Alkaloids	+ + +	+ + +
Saponnins	+ + +	+ ++
Phenols	+ + +	++
Cardiac Glycosides	+ + +	+ + +
Flavonoids	+ + +	+++
Steroids	+ + +	++
Anthraquinone Glycosides	+ + +	++
Terpenoids	+ + +	++

+ + + shows rich, while + + shows comparatively small amount

### Effect on rabbit jejunum

**Fig. 1:**
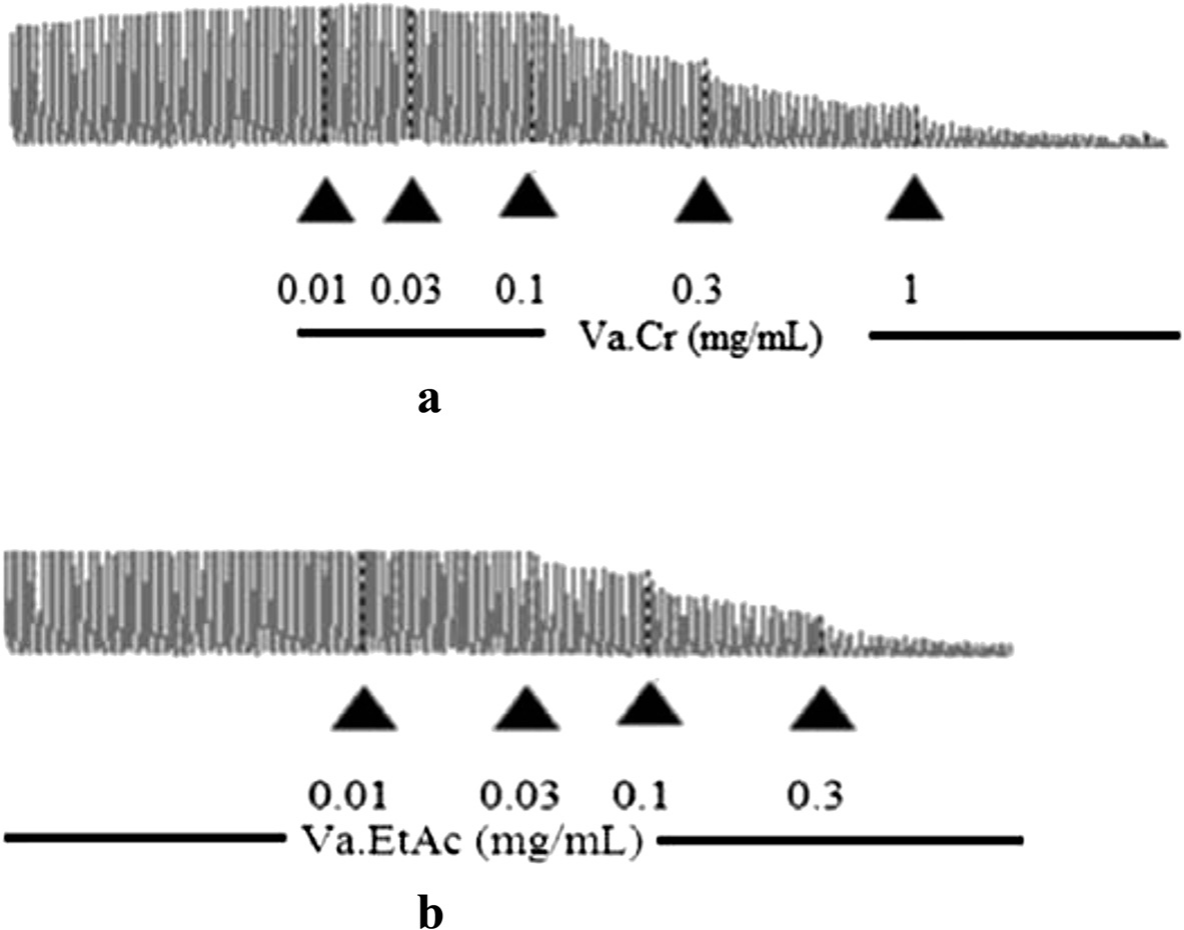
A typical tracing showing spasmolytic effect of **a** crude extract of *Viscum album* (Va. Cr) and **b** ethyl acetate fraction (Va. EtAc) on spontaneous contractions in isolated rabbit jejunum preparation

**Fig. 2:**
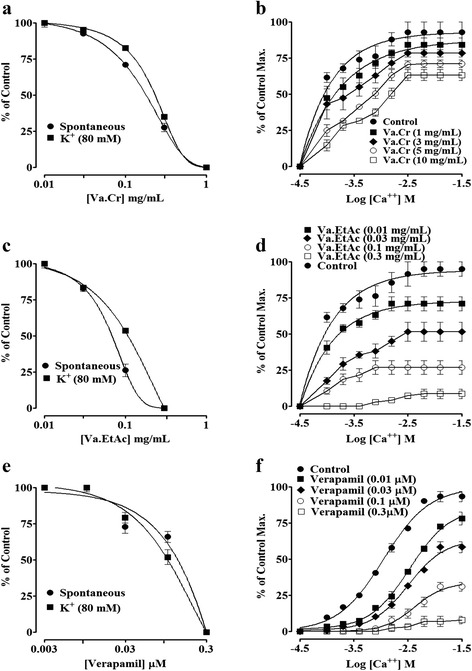
Concentration-response curves of; **a** the crude extract of *Viscum album* (Va. Cr), **c** Ethyl acetate fraction (Va. EtAc), and **e** verapamil on spontaneous and high K^+^ (80 mM)-induced contractions. **b**, **d** and **f** depict the effect on Ca^++^ concentration-response curves in the absence and presence of different concentrations of Va. Cr, Va. EtAc and verapamil, respectively, in isolated rabbit jejunum preparations. Values shown are mean ± SEM (*n* = 5–7)

Va. Cr displayed an excellent spasmolytic activity and inhibited the spontaneous contractions of rabbit jejunum (Fig. 1) with an EC_50_ value of 0.31 mg/mL (0.15–0.57) (Fig. 2). Similarly, Va. Cr relaxed the K^+^ (80 mM)-induced sustained contractions in a dose-dependent manner with an EC_50_ value of 0.62 mg/mL (0.3–0.95) (Fig. 2a). The CaCl_2_ curves were shifted rightward (Fig. 2b) similarly to that caused by verapamil (Fig. 2f) when the tissue was pretreated with Va. Cr (0.01–0.3 mg/mL). Va. EtAc was found even more potent than Va. Cr and relaxed the spontaneous and high K^+^-induced contractions of the isolated rabbit jejunum with EC_50_ values of 0.16 (0.11–0.23) and 0.22 mg/mL (0.15–0.3), respectively (Fig. 2c). It also shifted the concentration-response curves for CaCl_2_ concentration-dependently (0.1–0.3 mg/mL) to the right correspondingly to verapamil (Fig. 2f).

### Effect on rabbit aorta

**Fig. 3:**
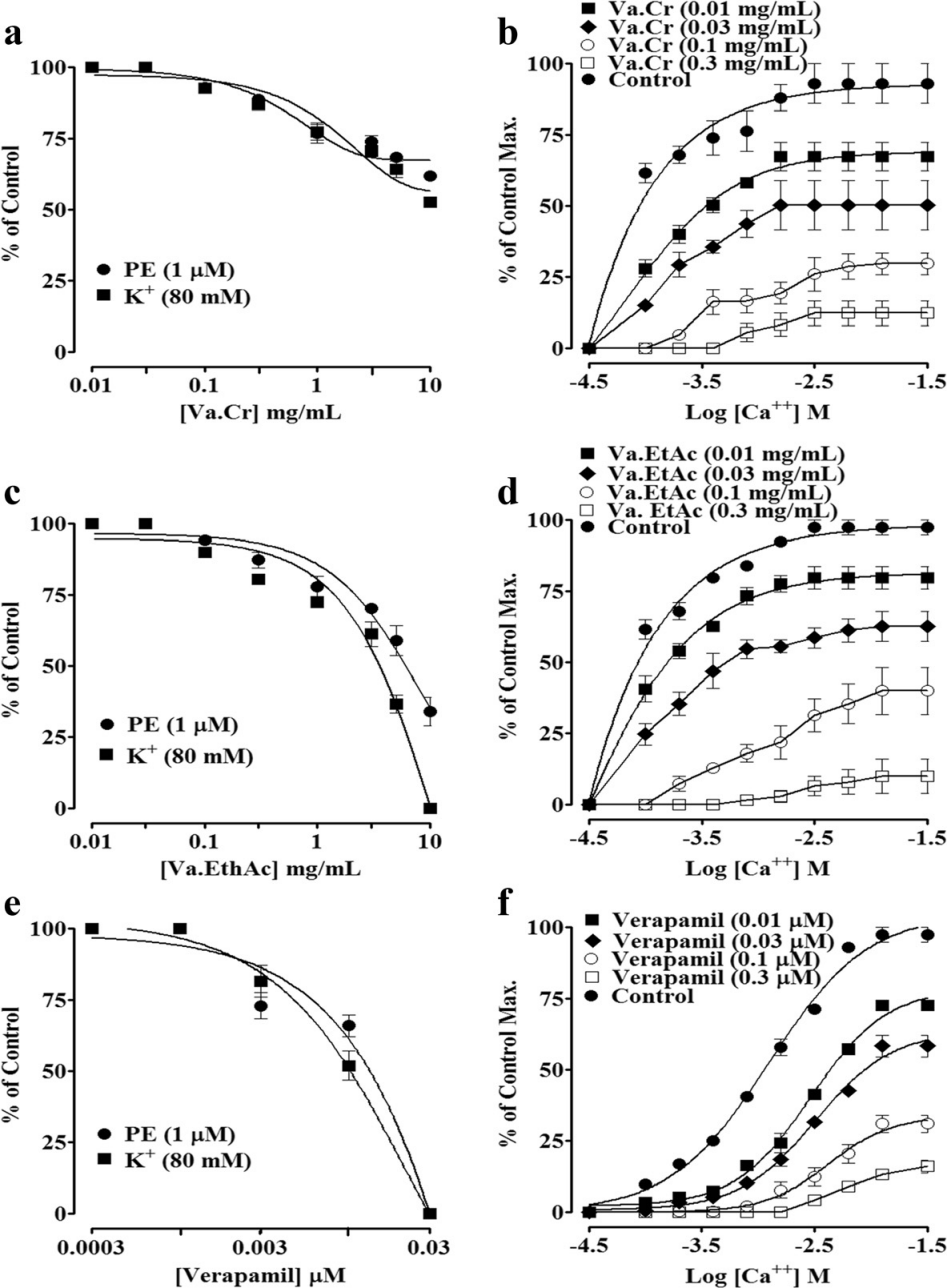
Concentration-response curves of; **a** the crude extract of *Viscum album* (Va. Cr), **c** Ethyl acetate fraction (Va. EtAc), and **e** verapamil on PE (1 μM) and high K^+^ (80 mM)-induced contractions. **b**, **d** and **f** depict the effect on Ca^++^ concentration-response curves in the absence and presence of different concentrations of Va. Cr, Va. EtAc and verapamil, respectively, in isolated rabbit aorta preparations. Values shown are mean ± SEM (*n* = 5–7)

Both Va. Cr and Va. EtAc partially inhibited PE (1 μM) and K^+^ (80 mM)-induced sustained contractions (Fig. 3), although these did not display any stimulatory activity on the base line tension. Pretreatment of the aortic ring with Va. Cr failed to shift CRCs rightward (Fig. 3a). Va. EtAc inhibited the contractions induced with high K^+^ with an EC_50_ value of 7.58 mg/mL (5.16–10) while it caused partial inhibition of PE (1 μM) sustained contractions (Fig. 3c). The CaCl_2_ CRCs obtained in a Ca^++^-free medium were shifted rightward (Fig. 3d) by the pretreatment of the aortic rings with Va. EtAc in a similar manner to that of verapamil (Fig. 3f).

## Discussion

Medicinally, *V. album* is used for gut and cardiac disorders especially hypertension by the local healers [7]. To the best of our knowledge and according to the literature search, there is no scientific evidence for its traditional uses in these disorders. Owing to the gastrointestinal relaxation properties, the crude extract derived from *V. album* was tested on the isolated preparation of rabbit jejunum. Such isolated preparations display spontaneous rhythmic contractions, which allow the direct evaluation of the relaxant (spasmolytic) effects of the samples without the use of an agonist (spasmogen) [20]. Va. Cr showed antispasmodic activity in a dose-dependent manner similarly to that of verapamil, which is considered as a standard calcium channel blocking agent [27]. Activation of the contractile elements of smooth muscles including those of the rabbit jejunum preparations is dependent on an increase in the concentration of cytoplasmic free Ca^++^ [14]. The cellular Ca^++^ is increased either by influx through voltage-dependent Ca^++^ channels or discharge from intracellular depots present in sarcoplasmic reticulum. The spontaneous movements of the intestine are regulated by the periodic depolarization and repolarization. The action potential emerges as a swift influx of Ca^++^ through voltage dependent Ca^++^ channels at the peak of depolarization [15]. Furthermore, the interchange of the intracellular and extracellular calcium stores also initiates the periodic depolarization and repolarization in jejunal tissues [28]. In the light of the above discussion, it is concluded that Va. Cr and Va. EtAc may inhibit the spontaneous contractions of the rabbit jejunum by interfering either with the Ca^++^ release from the Ca^++^ stores or with the Ca^++^ influx through voltage dependent Ca^++^ channels. It was reported previously that the chemical constituents occurring in various medicinal plants execute their spasmolytic activities through blockade of the Ca^++^ channels [20]. Preliminary phytochemical analysis indicated presence of flavonoids, saponins and tannins, which has further strengthen our findings as plant derived flavonoids [29], saponins [30] and tannins [31] have been found to possess CCB effect, which might be the active candidate (s) responsible for the spasmolytic effect of *V. album*.

Sustained contractions of the of the rabbit jejunum were induced by the introduction of a high concentration of K^+^ in order to see if the Va. Cr and Va. EtAc mediate the spasmolytic effect through inhibition of calcium entry. Va. Cr and Va. EtAc were added cumulatively, which relaxed the induced contraction in a concentration-dependent manner. This clearly suggested an inhibitory effect on the Ca^++^ entry. The high K^+^-induced contractions depend on Ca^++^ entry through voltage-dependent Ca^++^ channels [21] and substances capable of inhibiting such contractions are assumed to have a possible calcium entry blocking effect [32]. Therefore, the inhibition of K^+^ (80 mM)-induced contractions of rabbit jejunum by Va. Cr and Va. EtAc may reflect inhibitory effect on the Ca^++^ entry through voltage-dependent Ca^++^ channels. This hypothesis was further confirmed by the pre-incubation of the jejunal preparations with the extract, which caused a shift in the Ca^++^ concentration response curves to the right similarly to verapamil [33]. This rightward displacement may be due to the presence of phytochemicals like alkaloids and tannins in Va. Cr and Va. EtAc as these types of constituents have shown calcium entry blocking activity. The results of the current study clearly indicated that the extract of *V. album* possesses antispasmodic activity. It is possible that this activity of the extract may be due to the calcium entry blocking effect as CCB are considered useful antispasmodic agents [34]. The preliminary phytochemical investigations revealed the presence of tannins, alkaloids, saponins and flavonoids (Table 1), which are effective CCB agents [35]. Therefore, the spasmolytic activity of Va. Cr and Va. EtAc may possibly be due to the presence of the calcium channel antagonizing constituents.

The plant extract did not display vasoconstrictor effect when applied to vascular preparations at resting tension (data not shown). However, the cumulative addition of Va. Cr to the aortic rings that were precontracted with PE (1 μM) or high K^+^ partially inhibited the induced contractions. On the other hand, Va. EtAc caused partial inhibition of PE pre-contractions and complete inhibition of high K^+^-induced contractions. Interestingly, verapamil was also found more potent in inhibiting high K^+^-induced contractions, which is a typical property of the Ca^++^ channel blocking agents [36]. The antagonizing effect of Va. EtAc on high K^+^-induced contractions suggests verapamil like Ca^++^ entry blocking effect. High concentration of potassium as KCl causes significant contraction of blood vessels by depolarization of smooth muscle cells and increase of the Ca^++^ entry through voltage-sensitive L-type calcium channels [27, 32]. Pretreatment of tissues with Va. EtAc caused a rightward shift in the Ca^++^ CRCs obtained in the Ca^++−^free medium, similarly to verapamil, which provided a further confirmation of this possibility. In contrast, Va. Cr was expectedly without any prominent effect on Ca^++^ CRC. These results indicated that Ca^++^ entry blocking constituents are present in the crude extract, which exhibited a unique pattern of distribution in Va. Cr and Va. EtAc. Va. Cr contains those Ca^++^ entry blocking constituents which are specific for the intestinal smooth muscle and devoid of specificity for vascular smooth muscles. This can explain the possible mechanism of the spasmolytic effect of the extract and justify its medicinal use in hyperactive gut disorders, such as spasm and possibly diarrhea. The failure of Va. Cr to induce relaxation of the precontractions in vascular preparation may be due to the presence of other constituents, which has interfered the relaxation and need to be explored. Va. EtAc was more potent inhibitor of the high K^+^ precontractions in the intestinal smooth muscles, which may involve other relaxant constituents, in addition to the Ca^++^ entry blockers. Calcium channels are considered more active in vascular smooth muscle than others [37], so vascular relaxation induced by Va. EtAc can be pertained to the Ca^++^ entry blocking constituents. This provides a sound mechanistic base to the medicinal importance of the plant in cardiovascular disorders, particularly hypertension.

## Conclusions

This study showed that *V. album* possesses antispasmodic and vasodilatory effects that are induced through the blockade of Ca^++^ entry. Thus the current study provides a mechanistic evidence for the medicinal use of *V. album* in colic, diarrhea and hypertension. Further studies are needed to investigate the underlying molecular mechanisms.

## Abbreviations

CBB, Calcium Channels Blockade; CRCs, Ca^++^ Concentration-response Curves; EC_50_, Median Effective Concentrations; EDTA, Ethylenediaminetetraacetic Acid; PE, Phenylephrine; SEM, Standard Error Means; Va. Cr, *V. album* Crude Extract; Va. EtAc, *V. album* Ethyl Acetate Fraction; VDCs, Voltage-dependant Ca^++^ Channels
